# E-cadherin expression in the tongue epithelium of streptozotocin-induced diabetic rats: an exploratory study

**DOI:** 10.2340/aos.v84.43564

**Published:** 2025-05-13

**Authors:** Hassan Hamed Kaabi, Abdullah Mohamed Alsoghier, Islam Abdulrahim Alredah, Rayan Mohammed Alqahtani, Ibrahim Suliman Alsanie, Hanan Abdulgafour Balto

**Affiliations:** a Department of Oral Medicine and Diagnostic Sciences, College of Dentistry, King Saud University, Riyadh, Saudi Arabia; b Central Research Laboratory, Medical Research Unit on Experimental Animals, King Saud University; c Department of Restorative Dental Sciences, College of Dentistry, King Saud University, Riyadh, Saudi Arabia

**Keywords:** E-cadherin, epithelium, diabetes mellitus, diabetic complications, streptozotocin

## Abstract

**Objective:**

Little was found on the association between diabetes and its effect on epithelial intercellular adhesion. However, no study reported the association between hyperglycemia and E-cadherin-mediated cell adhesion in tongue epithelium. This study aimed to explore the potential impacts of hyperglycemia on the epithelial E-cadherin expression in the tongue’s epithelial tissue in streptozotocin (STZ)-induced diabetic rats.

**Material and methods:**

Twelve male Wistar albino rats were randomly allocated into control and STZ-induced diabetic groups. At the 5-week post-STZ injection, rats were euthanized, and the tongues were harvested and preserved in formalin. Epithelial thickness was assessed using hematoxylin and eosin (H&E) staining, while immunohistochemistry (IHC) was employed to analyze the expression of E-cadherin. Statistical analysis was performed using unpaired *t*-tests and two-proportion Z-tests, with a significance level determined at *p* < 0.05.

**Results:**

The results showed a significant reduction in epithelial thickness in the dorsal tongue of STZ-diabetic rats compared to the control group (*p* = 0.0173). Additionally, E-cadherin expression in the dorsal tongue epithelium was markedly weaker in the diabetic group than in the control (*p* < 0.0001).

**Conclusions:**

This exploratory study is the first to report that hyperglycemia reduces E-cadherin expression in the dorsal tongue epithelium, possibly contributing to oral epithelial alterations observed in diabetes. These findings not only highlight the potential diagnostic value of E-cadherin as a biomarker for oral complications in diabetic patients but also provide a foundation for future translational and clinical studies exploring therapeutic interventions targeting epithelial integrity in diabetes.

## Introduction

Diabetes mellitus, a prevalent chronic disease, presents a significant global health challenge. It is characterized by hyperglycemia resulting from insulin dependence (type I diabetes) or resistance (type II diabetes) [1, 2]. Its impact extends beyond its financial burden on healthcare services, encompassing complications such as peripheral vascular disease, retinopathy, nephropathy, and neuropathy [2].

The intricate relationship between metabolic disorders, particularly diabetes, and oral health complications has been a long-standing focus of research. Oral complications associated with diabetes can present as oral dryness, periodontal tissue destruction, dental caries, and opportunistic infections [2, 3]. Individuals with diabetes have an elevated risk of developing oral cancer and precancerous lesions [4]. Moreover, numerous mucosal alterations that have been linked to diabetes include traumatic ulcers [5], recurrent aphthous ulceration, geographic and fissured tongue and lichen planus [3, 5].

The epithelium is the first layer of the oral mucosa, and it is constantly subjected to high functional demands, necessitating frequent turnover, to provide a protective coating to the underlying structures [6]. Evidence reveals quantitative and qualitative alterations and impaired wound healing of oral epithelium in diabetic patients [7–9]. Hyperglycemia in diabetes reduces the expression of intercellular adhesion molecules in oral epithelial tissues, leading to dissociation of cell junctions and different epithelial abnormalities. Previous studies’ findings suggested that hyperglycemia is associated with the induced disruption of intercellular adhesion and delayed wound healing in gingival epithelial tissues, leading to the development and progression of periodontal disease in diabetes [10, 11].

E-cadherin (CDH1, also known as cadherin-1) takes center stage regarding intercellular adhesion molecules and wound healing of epithelial tissues. As a tumor suppressor and cadherin glycoprotein involved in the calcium-dependent adhesion in epithelial cells, E-cadherin is crucial in maintaining epithelial cell survival, morphology, and polarity, as well as tissue integrity and homeostasis [12, 13]. E-cadherin-mediated adhesion junctions are vital for cell sheets’ directional migration, a process essential in wound healing by epithelial cells [14]. Its intracellular domain recruits beta-catenin proteins, thereby limiting the destabilization of cell junctional complexes and maintaining epithelial barrier function. Pathological processes that interfere with this barrier are associated with hyperproliferative lesions and delayed epithelial wound healing [13–15].

Only a few studies investigated the effects of diabetes on the expression of E-cadherin. A recent study explored the impact of diabetes on E-cadherin-mediated intercellular junctions in corneal epithelial tissues. The findings revealed that diabetic keratopathy, characterized by recurrent epithelial erosion, fragile epithelium, and delayed wound healing, was associated with the dissociation of epithelial cell junctions. The significant decrease in the expression of E-cadherin was indicated to be an underlying cause of the epithelial alterations due to hyperglycemia [15]. Another study suggests that impaired intercellular adhesion molecules (e.g. E-cadherin) in gingival epithelial cells under hyperglycemic conditions are related to the development of periodontal disease [10].

Previous studies did not necessarily detail the effect of hyperglycemia on E-cadherin-mediated cell adhesion in the various oral epithelial tissues. Given the importance of diabetes and the need for further investigation of the oral epithelial abnormalities likely encountered by diabetic patients, the present study aimed to explore the potential impacts of hyperglycemia on the epithelial E-cadherin expression in the tongue epithelial tissue in streptozotocin (STZ)-induced diabetic rats. Understanding the influence of diabetes on the oral epithelium is crucial for preventing and treating diabetes-associated oral complications.

## Methodology

Ethical approval to conduct this study was obtained from the Research Ethics Committee at King Saud University (KSU-SE-22-44), and the study was registered at the College of Dentistry Research Center (CDRC No. FR 0650). The animal study was performed at the Central Research Laboratory (CRL), King Saud University, Saudi Arabia.

The handling of animals followed the guidelines for the care and use of animals for scientific purposes. The reporting of the animal experiment adhered to the ARRIVE guidelines 2.0 [16]. We utilized the ARRIVE checklist by providing detailed information on the experimental design, sample size determination, randomization, blinding during data collection, and animal welfare measures, ensuring transparency and reproducibility.

### Animals and induction of diabetes

In the current study, 12 male Wistar albino rats (aged 8–10 weeks) weighing approximately 220–240 g from the animal house at CRL were used. The sample size of 12 rats was determined by applying 95% power, 0.8 effect size, and alpha = 0.05 (G*Power version 3.1.9.4).

To minimize potential variability in results, we selected rats of similar age, weight, and baseline health conditions, and we standardized all experimental procedures and handling practices. This included maintaining uniform housing conditions (temperature, humidity, and light-dark cycle), providing identical diets and water access, and ensuring that the same trained personnel performed all treatments and sample collections to reduce variability.

Rats were randomly allocated into the STZ-induced diabetes and the control groups using an online random group generator (Research Randomizer) to ensure unbiased distribution. During the experimental procedures and analysis, blinding was maintained by coding the animal samples and group identities. The personnel responsible for data collection and analysis were unaware of the group allocations to prevent bias.

Ten days before the experiment, rats were housed individually in special clear-sided cages at controlled temperatures (22°C ± 2°C) and humidity (55% ± 5%), with a 12-h light-dark cycle. Rats were allowed access to food and water. The rats were weighed 1 day before the experiment (day 0), and their body weights were recorded. Blood samples were taken from the tail vein of each animal, and blood glucose levels were measured using a glucometer as a reference.

On day one of the experiment, all rats were fasted for 6 h before STZ treatment; only water was provided. Rats were given an intraperitoneal injection containing 95 mg/kg ketamine and 14 mg/kg xylazine. Once sedated, freshly prepared STZ (Santa Cruz Biotechnology) (32.5 mg/ml in sodium citrate buffer, pH 4.5) was injected intraperitoneally (in the caudal abdominal cavity using a sterile 25g needle) at 65 mg/kg (2.0 ml/kg) for the study group. The control group was injected intraperitoneally with an equal volume of citrate buffer (pH 4.5) [17]. Before injection, rats were held at a dorsal position in one hand, and the injection site was disinfected using a povidone-iodine swab. Rats were returned to their cages and provided with regular food. The control group was provided with regular water, whereas the STZ-treated rats were given 10% sucrose water for 2 days to avoid severe hypoglycemia. On experimental day 3, the 10% sucrose water was switched to regular water. On experimental day 5, all rats were fasting for 6–8 h, and the blood glucose concentration was measured from a tail vein blood sample using a One Touch basic blood glucose monitoring system [Contour Next ONE] to check hyperglycemia. Severe diabetes was confirmed in all STZ-injected rats 5 days post-injection (blood glucose levels >250 mg/dl) [17]. Blood glucose and weight were monitored twice a week during the experimental period. Rats were euthanized 5 weeks (day 35) after the beginning of the experiment using carbon dioxide inhalation. Death was confirmed by the absence of breathing movements and heartbeats, followed by decapitation [18].

### Tongue epithelial thickness measurement

Following euthanasia, the whole tongue was excised and preserved immediately in a 10% formalin solution for 48 h, followed by standard histological processing and paraffin embedding [19]. Sections of 5 µm thickness were obtained and stained with hematoxylin and eosin (H&E). Whole slide images (WSIs) were generated using a scanner (KF-PRO-005, KFBIO, China), at 40x magnification. Calibration was done before each scanning session, and images were stored on a dedicated server (Histo-app image management system, Saudi Arabia). WSIs (Tiff extension) were downloaded from the server for analysis. Three different samples from each group were used to determine the epithelium’s thickness. For each sample, 10 interspaced areas along the epithelium of the dorsal portion of the tongue were measured [20] using ImageJ software (NIH, Bethesda, MD, USA). Epithelial thickness was measured at the same site in control and STZ rats. The analysis of the samples was blinded and performed by a single experienced observer.

### Immunohistochemistry

Immunohistochemistry (IHC) was performed to investigate the expression of the E-cadherin protein. Sections of 3 µm of the paraffin blocks of tongue tissues were prepared on coated glass slides, which were dried overnight before being heated on a slide warmer for 2h. The slides were deparaffinized in xylene and rehydrated through graded concentrations of ethanol solution. Citrate buffer (pH 6) was used for heat-induced antigen retrieval. The E-cadherin primary antibody (Abcam, ab231303) was diluted 1:200 in an antibody diluent (Abcam) and incubated with slides overnight at 4°C. The slides were incubated with horseradish peroxidase (HRP)-conjugated secondary antibody (Abcam, ab6789), and counterstained with hematoxylin.

WSIs for the IHC slides were generated as described above. For the quantification, an expert oral pathologist employed the open-source bioimage analysis software (QuPath) [21]. Random regions of interest (ROIs) from the dorsal epithelial tongue were selected using a random number generator to ensure unbiased sampling. Fixed-size areas (300 × 300 pixels) were used to standardize the analysis across the IHC staining of diabetic and control groups. The analysis was performed on similar areas of the dorsal epithelial tongue in both groups to ensure comparability. The entire process was conducted under blinded conditions, with group identities concealed until all data were collected and processed. Subsequently, positive cell detection was conducted in QuPath. This involved using the cell detection function to identify cells within the ROIs that met a specified pixel intensity threshold range between 0.05 and 0.10, effectively minimizing background noise and excluding irrelevant signals. Then, the total number of positive and negative detections within the ROIs was extracted from QuPath.

### Statistical analysis

The means of two independent groups were compared using an unpaired *t*-test in GraphPad Prism [10.2.3, 403]. Quantitative data are presented as means (±SD). A two-proportion Z-test was used to compare the proportions of positive E-cadherin expression detected by immunohistochemistry between the control and diabetic groups. The significance level was set at 5%; any test result with a *p*-value less than 0.05 was considered significant.

## Results

One diabetic rat died in the 5^th^ week after the STZ injection. The cause of death was not investigated; however, it could be related to STZ toxicity or diabetic complications [22]. Given that the rat died at the end of the experiment, a replacement was not feasible. Starting from day 5 post-STZ injection until the end of the experiment, the diabetic rats had increased blood glucose levels and decreased body weights compared with the control group (Table 1).

**Table 1:** Mean blood sugar levels and body weights for control and STZ-diabetic groups at different time points.

Group	Day 0 Mean ± SD	*P*	Day 5 Mean ± SD	*P*	Day 35 Mean ± SD	*P*
**Body weight (g)**						
Control	235.40 ± 9.9	0.9091	259.2 ± 10.2	0.0053^*^	312.4 ±19. 3	<0.0001^*^
Diabetic	235 ± 18.2		218.4 ± 24		189 ± 28.7	
**Blood sugar (mg/dl)**						
Control	91.5 ± 10.9	0.2376	87 ± 4.6	<0.0001^*^	94.4 ± 4.1	<0.0001^*^
Diabetic	83.2 ± 10.7		545 ± 64.1		553.4 ± 23.3	

SD: standard deviation.

(*) Significant difference (unpaired *t*-test).

**Figure 1:**
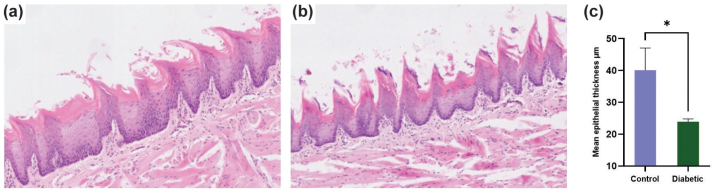
H&E staining of the dorsal tongue; (a) control and (b) diabetic rats (Magnification: 10X). (c) Mean epithelial thickness of the dorsal tongue in control and diabetic groups (n = 3; p = 0.0173); the comparison was made using an unpaired t-test.

**Figure 2:**
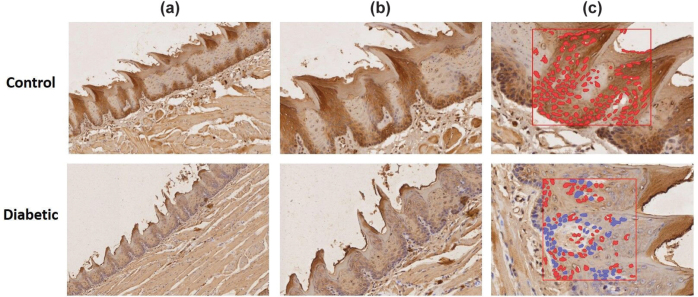
(a, b) IHC for E-cadherin in the epithelium of the dorsal tongue. Strong staining for E-cadherin was observed in the cytoplasm and membranes of epithelial cells from the control group, but was expressed at lower levels in the diabetic group. (c) Quantitative assessment using positive cell detection in QuPath, showing the control group with 100% positivity and the diabetic group showing less positivity at 70.5% (n = 3; p < 0.0001); the analysis was made using a two-proportion Z-test (Magnification: 10X A, 20X B, 40X C).

The results showed a reduced epithelial thickness of dorsal tongue in diabetic rats (mean = 23.9 ± 1.4 µm) compared with the control group (mean = 40.1 ± 7.07 µm), and the difference was statistically significant with a large effect size (*p =* 0.0173, Cohen’s d = 3.18) (Figure 1). The expression of E-cadherin in the dorsal tongue of rats using IHC demonstrated strong expression in the control group and weak expression in the diabetic group (Figure 2a, b). To validate the subjective interpretation of IHC data, the quantitative assessment of E-cadherin expression showed 100% positivity in the control group, compared to 70.5% in the diabetic group. A two-proportion Z-test confirmed a significant reduction in the diabetic group (*n* = 3; *p* < 0.0001, Cohen’s h = 1.15) (Figure 2c).

## Discussion

Little was found in the literature on the association between diabetes and the epithelial intercellular protein E-cadherin. A recent study found that chronic hyperglycemia reduces the expression of E-cadherin in the corneal epithelium [15]. In the oral cavity, the effect of hyperglycemia on E-cadherin was investigated in the gingival epithelium [10]. To our knowledge, the other oral epithelial tissues have not been thoroughly investigated. Therefore, the present work explored the potential impact of diabetes on the expression of E-cadherin in tongue epithelium in diabetic rats.

The study used rats due to their similarity to humans and advantages over other species. Their relatively low cost, ease of manipulation and ability to maintain a controlled environment are among these advantages. Furthermore, there is a remarkable similarity between the oral mucosa of rats and humans, which comprises the surface epithelial tissues and the underlying basal lamina [19].

The oral mucosa is lined with keratinized or non-keratinized squamous stratified epithelium, with keratinized epithelium found in masticatory mucosa such as the hard palate and gingiva, and non-keratinized epithelium covering the lining mucosa, including the buccal mucosa and soft palate. The dorsal surface of the tongue represents an area of specialized mucosa, characterized by keratinized epithelium and lingual papillae [23]. This unique anatomical and functional complexity makes the tongue highly susceptible to pathological changes, particularly in systemic conditions like diabetes. Previous studies have identified the tongue as a commonly affected site for oral lesions, including traumatic ulcers and oral cancer, in diabetic patients [5, 24]. However, limited research has focused on the tongue’s epithelial response to hyperglycemia. By investigating E-cadherin expression in the dorsal tongue epithelium, the current study addresses this gap and provides novel insights into how diabetes impacts epithelial integrity and intercellular adhesion in this unique oral tissue. These findings underscore the importance of the tongue as a model for studying diabetes-related oral alterations and pave the way for future research targeting this underexplored tissue.

Emerging evidence suggests that diabetes induces notable changes in the thickness and cellular morphology in different epithelial tissues [7, 11, 15, 25]. Morphologically, the present study only assessed the epithelial thickness of the dorsal tongue, which was significantly reduced compared with the control. This agrees with previous studies that demonstrated reduced oral and corneal epithelial thickness in diabetic rats and mice [15, 25, 26]. In contrast, studies on oral and corneal epithelium in diabetic rats observed no epithelial atrophy [26, 27]. This discrepancy might be related to differences in the epithelial type, animal age, diabetic induction method, and length of study.

The IHC analysis presently indicated a significant difference in E-cadherin expression between the control and diabetic groups. While intense E-cadherin staining was observed in the cytoplasm and membranes of epithelial cells in the control group, this expression was notably reduced in the diabetic group. This subjective assessment was supported by quantitative histomorphometry [28], which demonstrated a 100% E-cadherin positivity in the control group compared to 70.5% in the diabetic group. This finding was in line with previous studies, which suggest that hyperglycemia in diabetes is associated with the induced reduction of E-cadherin in epithelial tissues, leading to several diabetic complications such as hyperproliferative lesions, impaired epithelial wound healing, periodontal diseases, and recurrent corneal erosion [10, 13–15]. The E-cadherin intracellular domain recruits the β-catenin protein, which connects with α-catenin to mediate the anchoring of the actin cytoskeleton for stability [29]. Hyperglycemia in diabetes was suggested to decrease the binding between E-cadherin and b-catenin, leading to cytoplasmic accumulation of free β-catenin molecules, which translocate into the nucleus. Nuclear β-catenin can activate genes such as Snail, which suppresses the expression of E-cadherin, resulting in complete or partial loss of E-cadherin-mediated cell adhesion [15].

Diabetes-associated hyperglycemia is associated with proportional oral epithelial alterations often seen clinically. Hyperglycemia-induced reduction of E-cadherin in epithelial tissues is associated with periodontal diseases [10]. A study suggested that the epithelial-mesenchymal transition process, characterized by the downregulation of E-cadherin and upregulation of N-cadherin, may be involved in the pathogenesis of chronic periodontitis [30]. Additionally, reduced E-cadherin expression levels in the diabetic group were linked with invasive disease and possibly poor outcomes in patients with oral squamous cell carcinoma and, therefore, acted as a valuable clinical predictor for tumor behavior, treatment outcomes, and survival [31, 32].

The findings of this study are subject to some limitations. The mortality of one diabetic rat may have introduced bias by reducing the sample size and potentially affecting the generalizability of the results. Furthermore, the small sample size and the focus on a single type of oral epithelial tissue limit the applicability of the findings to other epithelial tissues. Additionally, the study did not investigate the mechanisms underlying the hyperglycemia-induced reduction in E-cadherin expression or determine the glycemic thresholds at which hyperglycemia triggers cellular changes in oral epithelial tissues. Consequently, further comprehensive translational and clinical work is needed to evaluate the qualitative and quantitative effects of hyperglycemia-induced alterations in E-cadherin expression across various oral epithelial tissues.

## Conclusions

In conclusion, this exploratory study demonstrates that hyperglycemia reduces E-cadherin expression in the dorsal tongue epithelium, a phenomenon often associated with oral epithelial alterations. These findings align with previous animal studies and provide further evidence of the detrimental effects of diabetes on epithelial intercellular adhesion. By emphasizing the impact of hyperglycemia on oral epithelial health, this study highlights the importance of future translational and clinical research to explore the diagnostic and therapeutic potential of targeting reduced E-cadherin in diabetes-associated oral complications. Such advancements could guide healthcare strategies to mitigate oral complications and improve outcomes for diabetic patients.

## Data Availability

The datasets generated and/or analyzed during the study are available from the corresponding author by reasonable request.
